# A gemcitabine sensitivity screen identifies a role for NEK9 in the replication stress response

**DOI:** 10.1093/nar/gku840

**Published:** 2014-09-12

**Authors:** Scott C. Smith, Aleksandra V. Petrova, Matthew Z. Madden, Hongyan Wang, Yunfeng Pan, Matthew D. Warren, Claire W. Hardy, Dong Liang, Elaine A. Liu, M. Hope Robinson, Soumon Rudra, Jie Wang, Shahrzad Ehdaivand, Mylin A. Torres, Ya Wang, David S. Yu

**Affiliations:** 1 Department of Radiation Oncology, Emory University School of Medicine, Atlanta, GA 30322, USA; 2 Department of Pathology, Emory University School of Medicine, Atlanta, GA 30322, USA

## Abstract

The Replication Stress Response (RSR) is a signaling network that recognizes challenges to DNA replication and coordinates diverse DNA repair and cell-cycle checkpoint pathways. Gemcitabine is a nucleoside analogue that causes cytotoxicity by inducing DNA replication blocks. Using a synthetic lethal screen of a RNAi library of nuclear enzymes to identify genes that when silenced cause gemcitabine sensitization or resistance in human triple-negative breast cancer cells, we identified NIMA (never in mitosis gene A)-related kinase 9 (NEK9) as a key component of the RSR. NEK9 depletion in cells leads to replication stress hypersensitivity, spontaneous accumulation of DNA damage and RPA70 foci, and an impairment in recovery from replication arrest. NEK9 protein levels also increase in response to replication stress. NEK9 complexes with CHK1, and moreover, NEK9 depletion impairs CHK1 autophosphorylation and kinase activity in response to replication stress. Thus, NEK9 is a critical component of the RSR that promotes CHK1 activity, maintaining genome integrity following challenges to DNA replication.

## INTRODUCTION

DNA replication is an essential process in all dividing cells and must be tightly regulated in order to preserve genome integrity. DNA replication is often impeded by DNA damage or replication blocks, and the resulting stalled replication forks are sensed and protected by a surveillance mechanism called the Replication Stress Response (RSR), a subset of the DNA damage response (DDR). The RSR plays an essential role in preventing the breakdown of stalled replication forks and accumulation of DNA structures that enhance recombination and chromosomal rearrangements, which cause genomic instability. Central to the RSR are the ataxia-telangiectasia and Rad3-related protein (ATR) checkpoint kinase and its downstream effector kinase checkpoint kinase 1 (CHK1) (1). ATR senses stalled replication forks as a consequence of fork uncoupling (2). When DNA polymerases stall, the MCM replicative helicases continue DNA unwinding ahead of the replication fork, leading to the generation of single-stranded DNA (ssDNA), which is then bound by the single-stranded binding protein replication protein A (RPA) to initiate the RSR. The ssDNA-RPA complex independently recruits ATR and its regulatory partner ATR interacting protein (ATRIP) (3) leading to the autophosphorylation of ATR at Thr-1989 (4,5) and the Rad17 clamp loader (6), which then loads the Rad9-Hus1-Rad1 (9-1-1) clamp complex onto DNA (7). Phosphorylated Rad9 recruits topoisomerase II beta binding protein 1 (TopBP1), which binds and activates phosphorylated ATR (8–10). Once activated, ATR phosphorylates numerous downstream substrates including the CHK1 kinase at Ser317 and Ser345 (11,12), which stimulates CHK1 activity, leading to autophosphorylation at Ser296 (13). Activated CHK1 in turn phosphorylates additional targets involved in DNA replication, DNA repair and cell-cycle checkpoints necessary to maintain genome integrity. There are likely many additional proteins that participate in the RSR.

Gemcitabine is an agent that induces replication blocks by direct incorporation into DNA as a terminal nucleoside analogue and through inhibition of ribonucleotide reductase, which depletes nucleotides required for DNA synthesis. Gemcitabine is widely used as a chemotherapeutic agent in a number of malignancies, including triple-negative breast cancer (TNBC), a highly aggressive and difficult to treat type of breast cancer characterized by lack of expression of estrogen receptor, progesterone receptor and amplification of human epidermal growth factor receptor 2 (HER2). Identifying genes that determine gemcitabine sensitivity could lead to the identification of novel RSR genes as well as the development of novel predictive biomarkers for outcome to gemcitabine treatment or novel therapeutic targets to be used as an adjunct to gemcitabine treatment to individualize patient treatment (14–16). As such, we completed a synthetic lethal screen using a siRNA library of nuclear enzymes in human TNBC cells to identify RSR genes that determine response to gemcitabine treatment.

Never in mitosis gene A (NIMA)-related kinase 9 (NEK9), which was identified in our gemcitabine sensitivity screen, is a member of the NEK family of serine/threonine kinases that are emerging as important regulators of the cell-cycle and checkpoint control (17,18). There are 11 mammalian homologues of *Aspergillus nidulans* NIMA, which was originally identified as a protein kinase essential for mitosis (19–23). Whereas NEK1, NEK6, NEK8 and NEK11 have been directly linked to the RSR (24–30), the role for other NEK family members, including NEK9, is less clear. NEK9 is important for mitotic progression by signaling centrosome separation and spindle assembly through phosphorylation of NEK6 and NEK7 (31–34) and NEDD1 (35). Depletion of NEK9 induces mitotic catastrophe by impairing mitotic checkpoint control and spindle dynamics (36). NEK9 also interacts with the facilitates chromatin transcription (FACT) complex to modulate interphase progression (37), suggesting that it may have a role in DNA replication. Finally, NEK9 is a putative ATM/ATR substrate (38). In this study, we show that NEK9 has a critical role in promoting RSR activities, including enhancing the activity of CHK1.

## MATERIALS AND METHODS

### Cell lines

MDA-MB-231 cells were maintained in Dulbecco's modified Eagle's medium (DMEM) (Gibco, Life Technologies, Grand Island, NY, USA) supplemented with 10% fetal bovine serum (FBS) (Gibco), while HEK 293T, U2OS and HeLa cells were maintained in DMEM media supplemented with 7.5% FBS. Cell lines were obtained from the American Tissue Culture Collection (ATCC) and grown in a humidified incubator at 37°C with 5% carbon dioxide.

### Transfections

siRNA transfections were performed using HiPerfect transfection reagent (Qiagen, Valencia, CA, USA) according to the manufacturer's instructions. Plasmid transfections were performed using Lipofectamine 2000 (Invitrogen) according to the manufacturer's instructions.

**siRNAs.** The following siRNAs were purchased from Dharmacon (Waltham, MA, USA):

NT: (ATGAACGTGAATTGCTCAATT)
ATR: (CCUCCGUGAUGUUGCUUGA)
ATRIP: (GGTCCACAGATTATTAGA)
CHK1: (CTGAAGAAGCAGTCGCAGT)
NEK9-1: (GGACUCAAUGAAUUCAAUA)
NEK9-2: (GGAAUCCUUCAUAGAGAUA)
NEK9-3: (AGACAAAGCCUCCUAUCGA)
NEK9-4: (GUAGUAACAUCACGAACCA)

### Gemcitabine sensitivity screen

MDA-MB-231 cells were transfected in 96-well plates using HiPerfect with 25 nM siRNA from a custom siGenome siRNA library (Thermo Scientific, Waltham, MA, USA) of 4024 siRNAs corresponding to 1006 unique human nuclear enzyme genes (pools of 4 siRNAs targeting a unique sequence of each gene) using a one gene per well format. Twenty-four hours later plates were split 1:4, and after another 24 h were treated with or without 5 μM gemcitabine (Hospira Inc., Lake Forest, IL, USA) for 72 h prior to assaying for cell proliferation using WST-1 reagent (Roche Diagnostics, Indianapolis, IN, USA). Each plate contained two positive controls (ATR and CHK1) and several negative controls non-targeting (NT). Plate-to-plate variability was controlled by normalizing the values on each plate to the average of the negative control values on that plate. Our ultimate analysis was based on the ratio of gemcitabine treated/untreated viability normalized to the NT control ratio. The log2 transformation of these normalized viabilities was also analyzed. Positive hits were defined as having a viability less than 0.7 or greater than 1.3, −log *P* > 1 (one-tailed student's *t*-test compared to NT control), and log2 transformation *z*-score greater less than −1.5 or greater than 1.5. Candidate gemcitabine sensitivity genes were further validated in a secondary screen using individual siRNAs to control for off-target effects. Validated hits demonstrated significant gemcitabine sensitization (normalized viability less than 0.8, *P* < 0.05) to two or more individual siRNAs.

### Immunoblotting

Cells were harvested in phosphate buffered saline (PBS). Cells were then lysed for 30 min on ice with 1% NP-40 lysis buffer containing: 200 mM NaCl, 1% NP-40 (Sigma-Aldrich, St. Louis, MO, USA), 50 mM Tris-HCl (pH 8.0) and supplemented with fresh protease inhibitors. A Bradford Assay was used to determine protein concentration, wherein 50 μg of protein was loaded into a sodium dodecyl sulphate-polyacrylamide gel electrophoresis (SDS-PAGE) gel, and transferred to a polyvinylidene difluoride (PVDF) membrane. Membranes were blocked using 5% non-fat dry milk in Tris-buffered saline and 0.1% Tween 20 (Sigma-Aldrich) (TBST), and primary antibodies were incubated in 5% bovine serum albumin (BSA) (Sigma-Aldrich) in TBST. Detection was performed using either the Odyssey system (LI-COR Biosciences, Lincoln, NE, USA), or Pierce ECL Substrate (Thermo Scientific, Rockford, IL, USA) on radiography paper.

### Cell viability assay

MDA-MB-231, MIA PaCa-2 or HeLa cells were transfected in 96-well plates using HiPerfect with 25 nM siRNA. Twenty-four hours later plates were split 1:4, and after another 24 h were treated with or without 5 μM gemcitabine (Hospira Inc., Lake Forest, IL, USA) for 72 h prior to assaying for cell proliferation using WST-1 reagent (Roche Diagnostics, Indianapolis, IN, USA). Each plate contained two positive controls (ATR and CHK1) and several negative controls (NT). Plate-to-plate variability was controlled by normalizing the values on each plate to the average of the negative control values on that plate. The ratio of gemcitabine treated/untreated viability normalized to the NT control ratio was determined.

### Colony formation assay

Twenty-four hours after transfection with 25 nM siRNA, 700 cells were seeded into 6-well plates in triplicate. Cells were allowed to rest over night and then treated for 24 h as indicated. Fresh media was replaced and cells were monitored until >50 cells per colony were visible by microscopy. Cells were fixed with 0.5% crystal violet in methanol.

### Antibodies

Primary antibodies were purchased as follows: anti-phospho-(Ser296)-CHK1 (rabbit polyclonal, #2349S), anti-phospho-(Ser317)-CHK1 (rabbit polyclonal, #2344S) and anti-RPA70 (rabbit polyclonal, #2267) were from Cell Signaling (Cell Signaling, Danvers, MA, USA); anti-NEK9 (mouse monoclonal, #sc-100401), anti-CHK1 (mouse, monoclonal, #sc-8408) and anti-OctA (FLAG) (mouse, monoclonal, #sc-51590) were from Santa Cruz Biotechnology (Dallas, TX, USA); anti-γ-H2AX (mouse monoclonal, #05–636) and anti-glyceraldehyde 3-phosphate dehydrogenase (GAPDH) (mouse monoclonal, #MAB374) were from Millipore (Temecula, CA, USA). Mouse and rabbit horseradish peroxidase-conjugated secondary antibodies (anti-mouse, #7076S; anti-rabbit, #7074S) were from Cell Signaling. Normal mouse immunoglobulin G (IgG) (N103) was from Calbiochem, Millipore. Anti-mouse IgG-Alexa Fluor 488 was from Invitrogen. Anti-rabbit IR dye (800) (#926–32213) was from LI-COR Biosciences.

### Immunoprecipitation

Cells were lysed in 10% glycerol, 150 mM NaCl, 50 mM Tris, pH 7.5, 0.5 mM dithiothreitol (DTT), 5 ug/ml leupeptin and 0.75% 3-[(3-Cholamidopropyl)dimethylammonio]-1-propanesulfonate hydrate (CHAPS) (Fisher Bioreagents, Thermo Fisher Scientific, Waltham, MA, USA). Protein supernatant was suspended in 0.375% CHAPS lysis buffer. Note that 3 mg of lysate was pre-cleared with 50% protein G beads, immunoprecipitated with antibodies against CHK1 or NEK9 or mouse IgG and bound to 50% protein G beads pre-washed in 0.375% CHAPS lysis for 4 h at 4°C on a rotator. The beads were subsequently washed five times with 0.375% CHAPS lysis buffer, and then boiled for 5 min at 100°C.

### *In vitro* CHK1 kinase assay

An *in vitro* CHK1 kinase assay was performed as previously described (39). Briefly, HeLa cell extracts were prepared by using NE-PER kit (Pierce) according to the manufacturer's instructions. Nuclear extracts (250 μg) were then mixed with 1 μg of CHK1 antibody (sc-7898; Santa Cruz) in the presence of 10 μl of a 50% (v/v) protein A-Sepharoseslurry (Invitrogen) in 250 μl of Buffer A (0.5% NP40, 1 mM Na3VO4, 5 mM NaF and 0.2 mM PMSF in PBS buffer) and gently rotated overnight at 4°C. The immune complexes were washed twice with Buffer A then twice with Buffer B [10 mM HEPES (pH 8.0), 50 mM NaCl, 10 mM MgCl2, 10 mM MnCl2, 1mM ATP and 1 mM DTT]. The kinase immunoprecipitate was incubated at 30°C for 30 min with 1 μg of CDC25C (Enzo) in 25 μl Buffer B containing 5 μCi γ-^32^P- ATP (Perkin Elmer, Akron, OH, USA). Samples were analyzed by 12% SDS-PAGE and the kinase activities determined by the incorporation of ^32^P into CDC25C protein using the PhosphorImager.

### Immunofluorescence imaging

Transfected U2OS cells were plated with poly-L-lysine (Cultrex, cat.# 3438-100-01) coated glass coverslips. Cells were plated at a density of 150 000 cells per well in a 6-well plate and allowed to rest over night. Cells were fixed with 3% paraformaldehyde in PBS for 10–15 min, permeabilized with PBS with 0.5% Triton-X-100 for 10 min and blocked with 5% BSA in PBS for 15 min at room temperature. Primary and secondary antibodies were diluted in 1% BSA in PBS and incubated at 37°C for 1 h. Mounting of the coverslips onto microscope slides was done with Vectashield Hardset Mounting Medium with 4-,6-diamidino-2-phenylindole (DAPI) (Vector Labs, Burlingame, CA, USA). Coverslips were visualized and imaged using a Zeiss Observer.Z1 inverted microscope and AxioCam camera with Axiovision software (Carl Zeiss Microscopy GmbH, Jena, Germany).

### Cell-cycle recovery assay

Cell-cycle recovery was performed as previously described (40,41). Briefly, U2OS cells were transfected with NT, ATR, ATRIP or NEK9 siRNA, treated 72 h later with 3 mM hydroxyurea (HU) for 20 h (arrested), washed and released into 0.5 μg/ml nocodazole (Fisher) for 10 h (released) to trap cells in mitosis. Both suspended and adherent cells were harvested and fixed in ice-cold 70% ethanol and DNA was stained with 25 μg/ml propidium iodide (Sigma-Aldrich) in PBS containing 100 μg/ml DNase free RNase A (Qiagen). DNA content was measured by flow cytometry using a BD FACS Canto II flow cytometer and then analyzed by FloJo software gating analysis tool (Tree Star).

### Cell-cycle synchronization

Forty-eight hours after plating, MDA-MB-231 or 293T cells were treated with 500 μM L-mimosine (MP Biomedicals) in media for 24 h, washed twice with PBS and released into media for 0, 6, 10 or 24 h. Harvested cells were fixed in ice cold 70% ethanol and DNA was stained with 25 μg/ml propidium iodide (Sigma-Aldrich) in PBS containing 100 μg/ml RNase A (Qiagen). DNA content was measured with a BD FACS Canto II flow cytometer and analyzed using FlowJo software. Another portion of harvested cells was lysed in 1% NP-40 buffer (200 mM NaCl, 1% NP-40, 50mM Tris HCl pH 8.0) freshly supplemented with protease inhibitors. SDS-treated lysates were separated on a 12% polyacrylamine gel, transferred onto PVDF and probed using NEK9 (Santa Cruz, sc-100401) and GAPDH (Santa Cruz, sc-25778) antibodies. Signals were detected with the Odyssey system.

## RESULTS

### A nuclear enzyme synthetic lethal screen identifies gemcitabine sensitivity genes in TNBC

**Figure 1.**
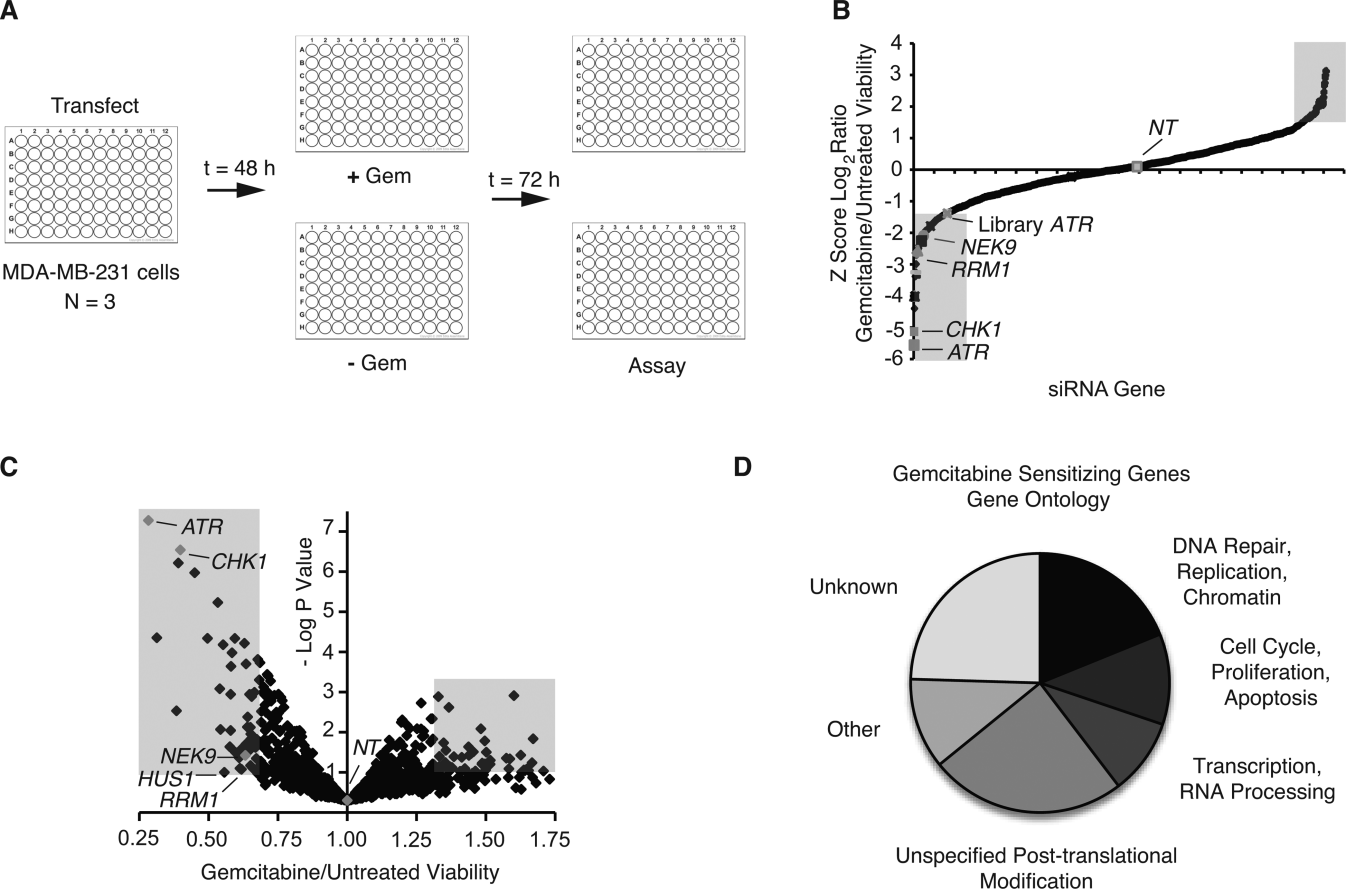
Synthetic lethal screen identifies gemcitabine sensitivity genes in TNBC. **(A)** Schematic representation of primary siRNA screen targeting 1006 unique human genes in MDA-MB-231 TNBC cells using a pool of four siRNAs per gene. **(B)** Summary of results of primary screen. The *z-*score of the log_2_ ratio of gemcitabine treated compared with untreated cell viability relative to a NT siRNA for each gene is shown. The shaded areas indicate positive hit criteria with a *z-*score <1.5 and >1.5. **(C)** Volcano plot of primary screen. The gemcitabine treated to untreated cell viability ratio and −log *P-*value for each gene relative to NT siRNA is shown. The shaded areas indicates positive hit criteria with a gemcitabine treated to untreated ratio <0.7 or >1.3 and a −log *P-*value >1. **(D)** Genes causing significant gemcitabine hypersensitivity upon silencing by known gene ontology function.

To identify genes involved in responding to replication blocks induced by gemcitabine treatment, we completed a synthetic lethal screen using RNA interference (RNAi) to identify genes that when silenced cause either sensitization or resistance to gemcitabine in human TNBC cells. We reasoned that gemcitabine sensitivity genes would likely be involved in the RSR. We therefore optimized a high-throughput assay using ATR and CHK1 siRNA as positive controls and a NT siRNA as a negative control with cell viability as a read-out (Figure 1A). The primary screen was completed in MDA-MB-231 TNBC cells, which consistently gave the highest signal-to-noise ratio among several tested TNBC cell types (data not shown). Briefly, cells were transfected with pools of four siRNAs targeting a unique sequence of each gene arrayed in a one gene per well format in 96-well plates. Forty-eight hours after transfection, cells were treated with or without 5 μM gemcitabine for 72 h prior to assaying for cell proliferation using WST-1 reagent. Each plate contained ATR, CHK1 and NT controls, and plate-to-plate variability was controlled by normalizing the values on each plate to the average of the negative control values on that plate. Western blot analysis confirmed knockdown of the ATR and CHK1 positive controls 48 h after transfection in MDA-MB-231 cells (Supplementary Figure S1). We completed three replicas of the primary screen using a library of 4024 siRNAs, corresponding to four unique siRNA duplexes, targeting each of 1006 unique human genes (Figure 1B and C, and Supplementary Table S1). The library consisted predominantly of nuclear enzymes, which we reasoned were more likely to function directly in the RSR and be targetable for future translational application. Hits met three criteria. Sensitization hits were defined as genes with a gemcitabine treated to untreated viability ratio of <0.7, a −log *P-*value > 1 and a *z*-score of log_2_ gemcitabine treated to untreated viability of <−1.5 (Figure 1B and C). The siRNA library ATR, with a viability of ∼0.7, served as a marker for these criteria. Gemcitabine resistance hits were defined as genes with a gemcitabine treated to untreated viability ratio of >1.3, a −log *P-*value of >1 and a *z*-score of log_2_ gemcitabine treated to untreated viability of >1.5 (Figure 1B and C). From our library of 1006 nuclear enzymes, we identified 53 gemcitabine sensitization genes and 33 gemcitabine resistance genes (Supplementary Tables S2 and S3). Of the 53 gemcitabine sensitization genes, 18 are linked to the RSR (Supplementary Table S2), including well-characterized ATR-signaling pathway genes CHK1, RAD1, HUS1 and CDC25A, demonstrating that our screen can yield RSR genes that determine gemcitabine sensitivity. Genes known to function in cell cycle, proliferation, apoptosis, RNA processing, transcription and post-translational modification were also identified (Figure 1D).

### NEK9 depletion causes replication stress hypersensitivity

**Figure 2.**
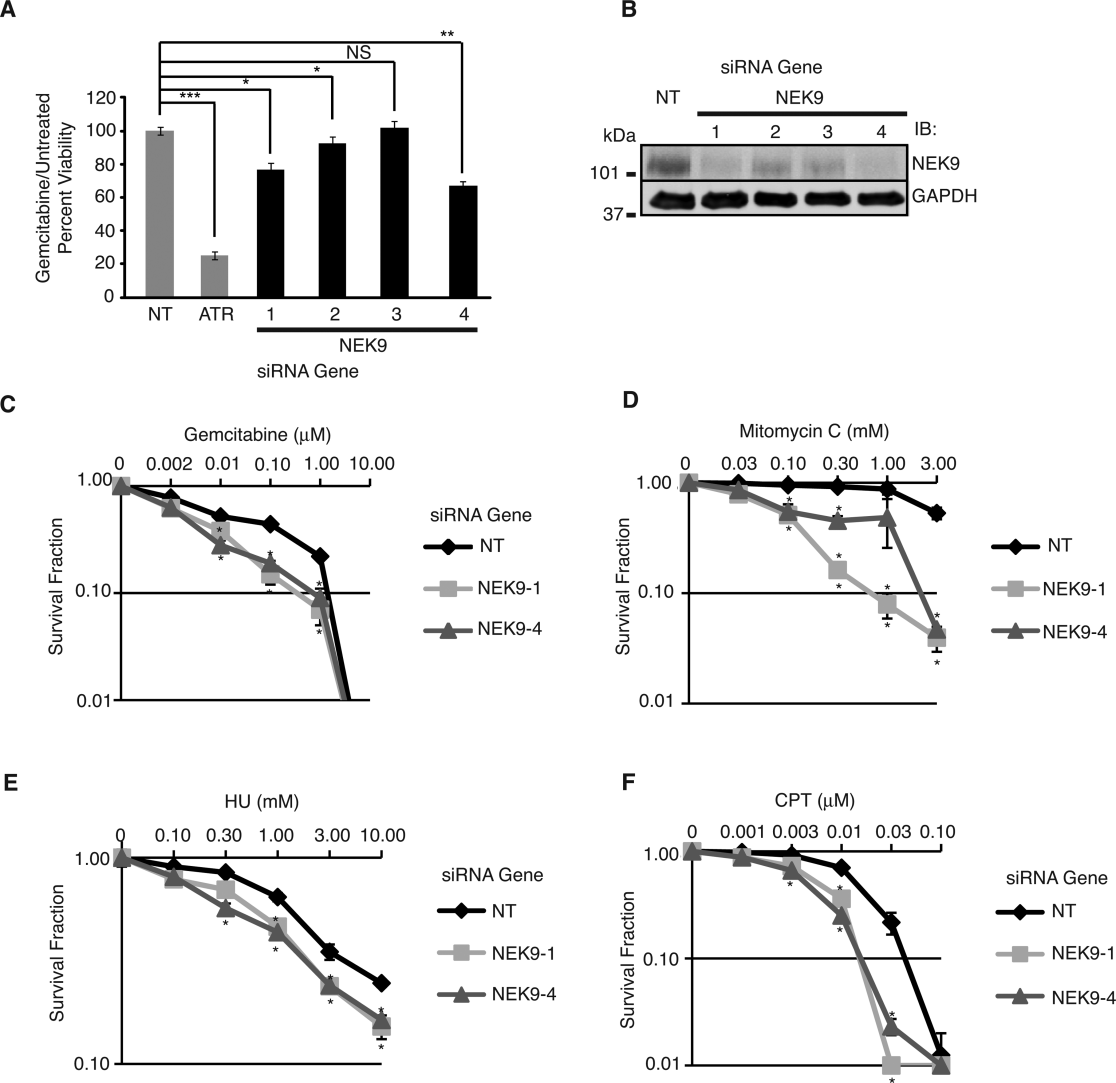
NEK9 depletion causes replication stress hypersensitivity. **(A)** MDA-MB-231 cells were transfected with NT, ATR or NEK9 siRNA, split 1:4 24 h later, and treated 24 h later with or without 5 μM gemcitabine for 72 h prior to assaying for cell viability. Gemcitabine treated to untreated cell viability relative to NT siRNA is shown. **(B)** Western blot analysis demonstrating efficiency of NEK9 knockdown with indicated siRNAs. **(C** and **D)** MDA-MB-231 cells transfected with NT or NEK9 siRNA were seeded for colony formation and treated with indicated concentrations of gemcitabine **(C)**, mitomycin-C **(D)**, HU **(E)** or camptothecin **(F)** for 24 h (h). Surviving colonies were counted 8–12 days later. Survival fraction of colonies from treated versus untreated cells is indicated. For **(A, C, D)**, mean and standard deviation from at least two replicas is shown. *P < 0.05; **P < 0.01; ***P < 0.001.

Twenty-three of our gemcitabine sensitization hits were identified in previously published DNA damage sensitivity screens (41–49) and seven are putative ATM/ATR substrates (38) (Supplementary Table S2). We utilized these criteria to validate 20 of the 53 hits not already characterized in the RSR in a secondary screen for cell viability using deconvoluted individual siRNAs to confirm their gemcitabine sensitivity and eliminate false positives due to off-target effects. Thirteen of these genes induced gemcitabine sensitivity in at least two out of four siRNAs tested. We selected NEK9 for further follow-up as three of four siRNAs targeting NEK9 caused gemcitabine hypersensitivity using cell viability as a read-out (Figure 2A), NEK9 was identified as a putative ATM/ATR substrate (38) and NEK9 was found to potentially interact with a number of DDR and DNA replication proteins through a mass spectrometry screen (50). Western blot analysis confirmed decreased levels of NEK9 following siRNA knockdown (Figure 2B), which were associated with gemcitabine sensitivity. A similar gemcitabine hypersensitivity was observed following NEK9 knockdown in MIA PaCa-2 human pancreatic cancer cells (Supplementary Figure S2A and B) and HeLa cervical cancer cells (Supplementary Figure S2C), indicating that gemcitabine hypersensitivity following NEK9 knockdown is not cell-type specific. We also determined the gemcitabine sensitivity of NEK9 depleted cells using a colony formation assay. MDA-MB-231 cells with NEK9 knockdown demonstrated a significantly reduced percentage of surviving colonies following a 24-h pulse of gemcitabine in a dose-dependent manner compared to a NT control (Figure 2C), confirming the gemcitabine hypersensitivity of NEK9 depleted cells observed with WST-1 reagent. NEK9 knockdown also caused hypersensitivity to mitomycin C, a DNA cross-linking agent (Figure 2D), HU, an inhibitor of ribonucleotide reductase (Figure 2E) and camptothecin, a topoisomerase I inhibitor (Figure 2F), suggesting that NEK9 responds generally to replication stress.

### NEK9 depletion leads to accumulation of DNA damage and RPA70 foci

**Figure 3.**
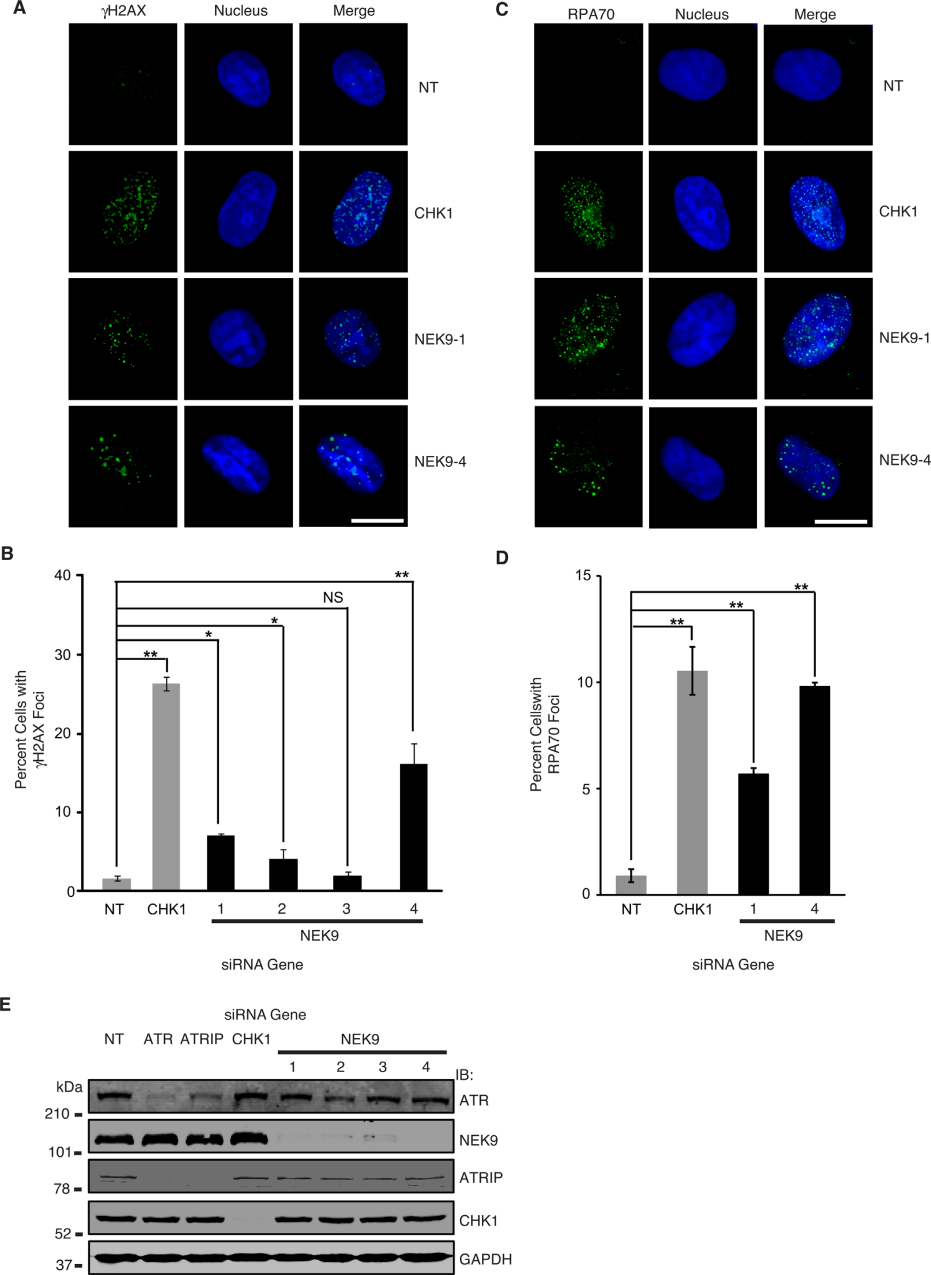
NEK9 depletion leads to accumulation of DNA damage and RPA70 foci. **(A)** U2OS cells were transfected with NT, CHK1 or NEK9 siRNA and processed 72 h later for γH2AX staining by indirect immunofluorescence. Representative images are shown. Scale bar indicates 10 μm. **(B)** The percentage (mean and standard deviation) of γH2AX positive cells (≥12 foci per cell) is shown. **(C)** U2OS cells were transfected with NT, CHK1 or NEK9 siRNA and processed 72 h later for RPA70 staining by indirect immunofluorescence. Representative images are shown. Scale bar indicates 10 μm. **(D)** The percentage (mean and standard deviation) of RPA70 foci positive cells (≥12 foci per cell) is shown. **(E)** Western blot analysis demonstrating efficiency of ATR, ATRIP, CHK1 and NEK9 knockdown with indicated siRNAs 72 h after transfection in U2OS cells. For **(B** and **D)** *P < 0.05; **P < 0.01; ***P < 0.001.

To validate NEK9 as a RSR protein, we examined for phosphorylation of H2AX Ser139 (γH2AX), an early marker for DNA damage, following NEK9 knockdown in U2OS human osteosarcoma cells (Figure 3E). In the absence of exogenous damage, stalled replication forks are expected to collapse, thereby leading to the accumulation of γH2AX. Similar to CHK1 knockdown, three of four siRNAs targeting NEK9 induced spontaneous accumulation of γH2AX foci compared to a NT control (Figure 3A and B). Moreover, NEK9 depletion in cells resulted in the spontaneous accumulation of RPA70 foci (Figure 3C and D), implying that NEK9 limits the amount of ssDNA available for RPA binding at stalled replication forks. Collectively, these results suggest that NEK9 functions to prevent the breakdown of stalled replication forks.

### NEK9 depletion impairs recovery from replication arrest

**Figure 4.**
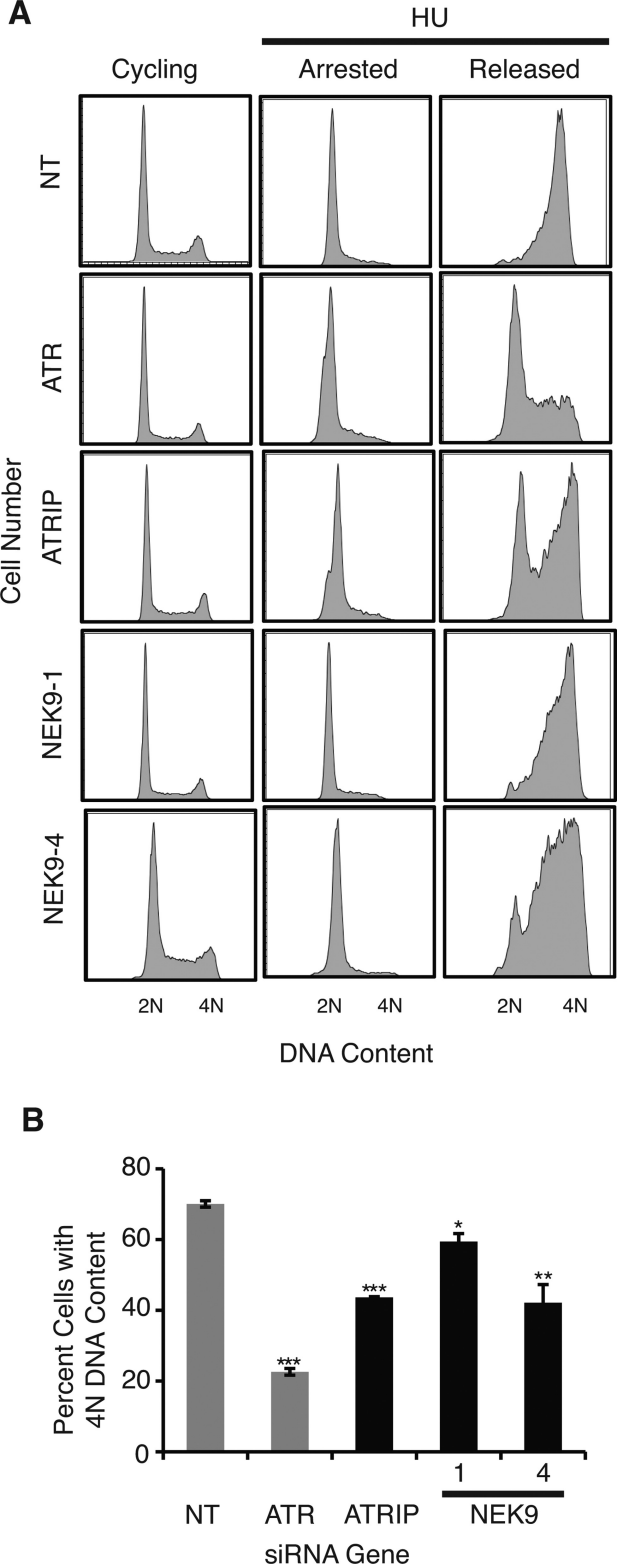
NEK9 depletion impairs recovery from replication stress. **(A)** U2OS cells were transfected with NT, ATR, ATRIP or NEK9 siRNA, treated 72 h later with 3 mM HU for 20 h (arrested), washed and released into nocodazole for 10 h (released). DNA content was analyzed by flow cytometry. **(B)** The percentage (mean and standard deviation) of cells that completed DNA synthesis in three replicate experiments is shown. *P < 0.05; **P < 0.01; ***P < 0.001.

To determine whether NEK9 is required for recovery from replication arrest, the ability of NEK9 depleted cells to recover from transient replication fork arrest was assessed using cell-cycle recovery by flow cytometry. Seventy-two hours following transfection (Figure 4A, cycling), U2OS cells were treated with HU for 20 h to arrest cells in early S-phase (Figure 4A, arrested). Cells were washed and released into nocodazole to measure cell-cycle progression following replication arrest (Figure 4A, released). Ten hours after removing HU, U2OS cells treated with a NT siRNA progressed through S-phase and accumulated 4N DNA content. In contrast, cells treated with ATR, ATRIP or NEK9 siRNA showed delayed S-phase progression (Figure 4A, released, and B), suggesting that NEK9 is required for recovery from replication arrest.

### NEK9 protein levels increase in response to replication stress

**Figure 5.**
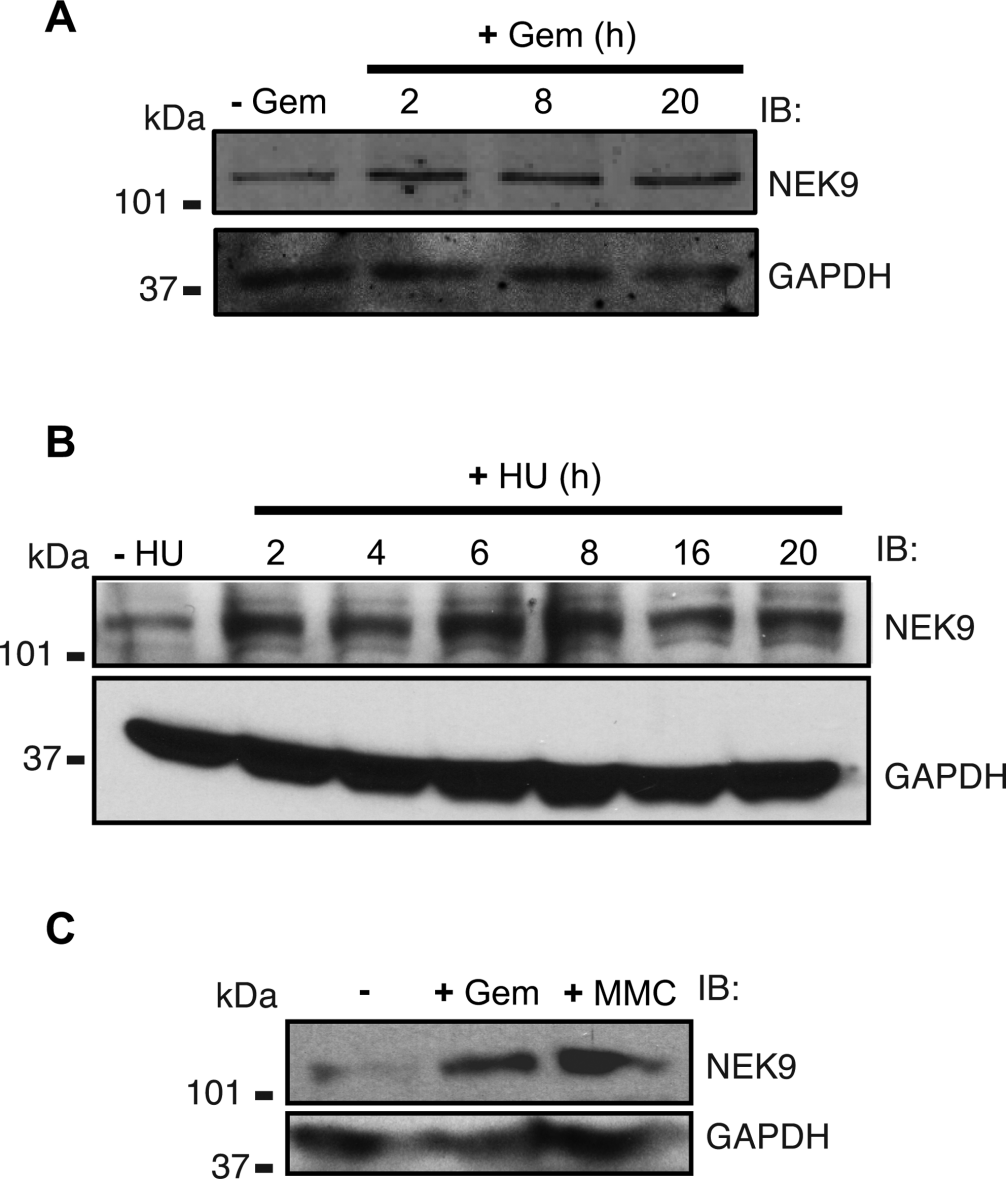
NEK9 protein levels increase in response to replication stress. **(A)** Western blot analysis of lysate from MDA-MB-231 cells treated with 1 mM gemcitabine for the indicated times. **(B)** Western blot analysis of lysate from HEK 293T cells treated with 3 mM HU for the indicated times. **(C)** Western blot analysis of lysate from HEK 293T cells treated with 1 mM gemcitabine or 3 uM mitomycin C for 6 h.

RSR proteins by definition respond to replication stress. To determine how NEK9 is regulated by replication stress, protein levels of NEK9 in MDA-MB-231 and HEK 293T cells following treatment with replication stress agents were determined. NEK9 protein levels increased in MDA-MB-231 cells 2 h after gemcitabine treatment and remained elevated up to 20 h after treatment (Figure 5A). NEK9 protein levels also increased in HEK 293T cells after HU, gemcitabine and mitomycin C treatment (Figure 5B and C), providing further support for our previous observation that NEK9 responds generally to replication stress. No significant increase in NEK9 protein levels was observed after synchronizing cells in S-phase (Supplementary Figure S3A–D), implying that the increase in NEK9 protein level observed following replication stress does not result from accumulation of cells in S-phase.

### NEK9 interacts in a complex with CHK1

**Figure 6.**
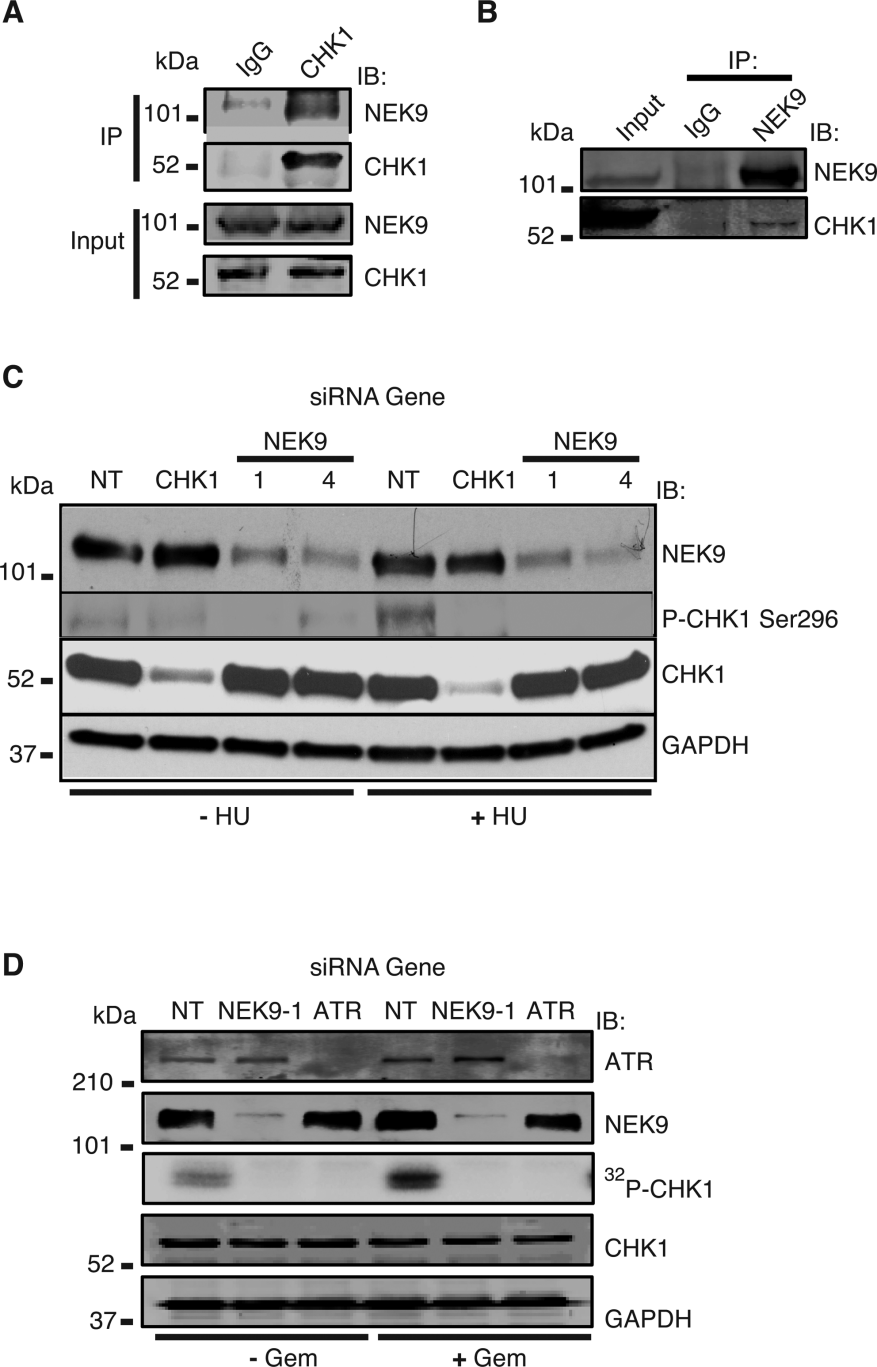
NEK9 complexes with and regulates the activity of CHK1. **(A** and **B)** Endogenous NEK9 or CHK1 was immunoprecipitated from cell lysates. Immunocomplexes were washed, separated by SDS-PAGE and immunoblotted with antibodies against NEK9 or CHK1. **(C)** NEK9 depletion impairs CHK1 autophosphorylation in response to replication stress. HeLa cells were transfected with NT, CHK1 or NEK9 siRNA, and treated with 3 mM HU for 6 h. Cell lysates were separated by SDS-PAGE, and immunoblotted with antibodies against NEK9, P-CHK1 Ser296, CHK1 and GAPDH. **(D)** NEK9 depletion impairs CHK1 kinase activity. CHK1 was purified from HeLa cells transfected with NT, ATR or NEK9 siRNA, treated with or without 1 mM gemcitabine for 3 h, incubated in an *in vitro* kinase reaction with ^32^P, and processed by autoradiography. The reaction mixtures were separated by SDS-PAGE and immunoblotted with antibodies against ATR, NEK9, CHK1 and GAPDH.

To gain insight into how NEK9 functions in the RSR, we searched for potential interacting partners in the RSR. A high-throughput mass spectrometry analysis of purified NEK9 previously identified its putative interaction with a number of proteins involved in the DDR or DNA replication including CHK1, KU70, KU80, RFC3, RRM1 and MCM5 (50). Given the central role of CHK1 in mediating gemcitabine sensitivity and recovery from replication stress, we explored its potential interaction with NEK9. Co-immunoprecipitation of endogenous CHK1 in 293T cells pulled down endogenous NEK9, and co-immunoprecipitation of endogenous NEK9 in cells pulled down endogenous CHK1 (Figure 6A and B), implying that the two proteins interact in a complex.

### NEK9 depletion impairs CHK1 autophosphorylation and kinase activity in response to replication stress

The interaction of NEK9 with CHK1 suggests that NEK9 may function in the CHK1 signaling pathway in response to replication stress. NEK9 knockdown significantly reduced CHK1 Ser296 autophosphorylation but not total CHK1 levels in response to HU treatment (Figure 6C), suggesting that NEK9 functions to control CHK1 activity in response to replication stress. As ATR directly phosphorylates and activates CHK1, we also examined cells for phosphorylation of CHK1 Ser317. No significant defect in CHK1 Ser317 phosphorylation after HU treatment was observed following NEK9 knockdown compared to a NT control (Supplementary Figure S4A) implying that NEK9 does not regulate ATR-dependent phosphorylation of CHK1. To determine if NEK9 regulates the kinase activity of CHK1, we performed an *in vitro* kinase assay using CHK1 itself and purified CDC25C as substrate. NEK9 depletion impaired CHK1 kinase activity in response to both gemcitabine and HU treatment (Figure 6D and Supplementary Figure S4B), implying that NEK9 regulates CHK1 kinase activity in response to replication stress.

## DISCUSSION

In this study we employed a synthetic lethal approach to identify novel RSR genes critical for determining gemcitabine sensitivity in TNBC. Among the 1006 unique nuclear enzymes in our primary siRNA library, we identified 53 gemcitabine sensitization genes and 33 gemcitabine resistance genes. As might be expected in a synthetic lethal screen using gemcitabine, which induces replication blocks, a large number of genes identified in our screen are linked to the RSR. Genes involved in cell-cycle, proliferation, apoptosis, transcription and RNA processing, including NEK9, were also identified, suggesting that gemcitabine sensitivity is mediated by diverse cellular processes. We validated NEK9 as a gemcitabine sensitivity gene and further found that depletion of NEK9 in cells results in replication stress hypersensitivity, spontaneous accumulation of DNA damage and RPA70 foci and an impairment in recovery from replication arrest. We also found that NEK9 protein levels in cells increases in response to replication stress. Finally, we found that NEK9 complexes with CHK1 and that NEK9 depletion in cells impairs CHK1 Ser296 autophosphorylation and kinase activity in response to replication stress. These findings reveal a novel function for NEK9 as a RSR protein, which promotes the activity of CHK1 in response to challenges to DNA replication.

NEK9 has a well-established function in mitotic progression by mediating early centrosome separation and spindle assembly through its downstream targets, NEK6 and NEK7, which in turn direct the recruitment of the kinesin EG5 to centrosomes as well as NEDD1 (31–34), contributing to γ-tubulin recruitment to centrosomes (35). NEK9 also interacts with the FACT complex to modulate interphase progression through an unknown mechanism (37). We now show that NEK9 has an additional function in activating CHK1 in response to replication stress, which may explain at least in part the replication stress hypersensitivity, spontaneous accumulation of DNA damage and RPA to foci and impairment in cell-cycle recovery observed following NEK9 depletion. In response to DNA damage, CHK1 is activated by ATR-dependent phosphorylation at Ser317 and Ser345, which is required for CHK1 autophosphorylation at Ser296 (51). Our observation that NEK9 depletion in cells impairs CHK1 Ser296 autophosphorylation and kinase activity but not CHK1 Ser317 phosphorylation in response to replication stress is consistent with a previous report that NEK9 does not affect CHK1 Ser345 phosphorylation (52) and suggests that NEK9 activates CHK1 downstream of ATR-dependent phosphorylation of CHK1. Indeed, it has been reported that CHK1 can be activated by a hypothetical trans-regulatory protein independent of CHK1 Ser345 phosphorylation (53). A further understanding of how NEK9 activates CHK1 will require identifying its substrate(s), which could include CHK1, claspin, which can activate CHK1 independently of ATR (54), and other associated proteins.

A role for NEK family members in regulating the RSR has previously been demonstrated for NEK1, NEK6, NEK8 and NEK11. NEK1 regulates the stability of the ATR-ATRIP complex and primes ATR for efficient DNA damage signaling, including leading to CHK1 activation (24,25,27,52). NEK6, which is a NEK9 substrate, is also a CHK1 substrate and is involved in the G2/M checkpoint (28). NEK8 physically and functionally interacts with ATR and CHK1 at the replication fork to prevent the accumulation of DNA damage induced by replication stress (29). NEK11 is a CHK1 substrate and promotes the G2/M checkpoint by phosphorylating CDC25A thereby targeting it for degradation (30). Our results extend these ideas and now demonstrate that NEK9 is also a component of the RSR that regulates the activity of CHK1 and support a paradigm for the NEK family of kinases in directing RSR activities.

The role of NEK9 in activating CHK1 in response to replication stress may not be exclusive of NEK9's activities in mitotic progression. CHK1 phosphorylation is also required for the G2/M checkpoint and for mitotic progression (55). CHK1 also negatively regulates PLK1 (56) and CDK1 (57), which are involved in NEK9 activation, suggesting possible negative feedback regulation, perhaps in a context-dependent manner mediated by cell-cycle phase or localization. In this regard, the regulation of NEK9 in response to genotoxic stress is still not clear. Our data suggest that NEK9 protein levels increase in response to replication stress, which may result from changes in NEK9 expression or stability. As the activity of PLK1 is inhibited by DNA damage (58), it is likely that the increase in NEK9 protein levels in response to replication stress is regulated by an alternative mechanism. One possibility is that NEK9 is regulated directly by ATR as NEK9 has been reported to be a putative ATM/ATR substrate (38).

Finally, our identification of NEK9 and other novel gemcitabine sensitivity genes through our synthetic lethal screen in human TNBC cells could lead to the development of novel therapeutic approaches for the treatment of TNBC and other malignancies treated with gemcitabine. TNBCs with decreased expression of NEK9 or other gemcitabine sensitivity genes may be more susceptible to gemcitabine treatment through synthetic lethality and thus with future validation, NEK9 expression could be utilized as a predictive biomarker to individualize TNBC patient treatment. In addition, for patients with high NEK9 expression who otherwise might be predicted to have poor response to gemcitabine treatment, NEK9 could function as a novel therapeutic target to be combined with gemcitabine treatment. As our RNAi library included nuclear enzymes, rational strategies using small molecule inhibitors could be developed targeting the novel gemcitabine sensitivity genes identified in our screen.

## SUPPLEMENTARY DATA

Supplementary Data are available at NAR Online.

SUPPLEMENTARY DATA
