# Supplementary material for: A gemcitabine sensitivity screen identifies a role for NEK9 in the replication stress response
Source: Nucleic Acids Res. 2014 Sep 12;42(18):11517–27. doi: 10.1093/nar/gku840 (PMC4191414; doi:10.1093/nar/gku840)
Supplement: SUPPLEMENTARY DATA [file supp_42_18_11517__index.html]

A gemcitabine sensitivity screen identifies a role for NEK9 in the replication stress response — SUPPLEMENTARY DATA 

# A gemcitabine sensitivity screen identifies a role for NEK9 in the replication stress response

## SUPPLEMENTARY DATA

**Files in this Data Supplement:**

- SUPPLEMENTARY DATA
- SUPPLEMENTARY DATA
