# Supplementary material for: A gemcitabine sensitivity screen identifies a role for NEK9 in the replication stress response
Source: Nucleic Acids Res. 2014 Sep 12;42(18):11517–27. doi: 10.1093/nar/gku840 (PMC4191414; doi:10.1093/nar/gku840)
Supplement: SUPPLEMENTARY DATA [file supp_gku840_nar-02238-h-2014-File008.pdf]

Supplementary Information

A Gemcitabine Sensitivity Screen Identifies a Role for NEK9 in the Replication Stress Response

Scott C. Smith[1], Aleksandra V. Petrova[1†], Matthew Z. Madden[1†], Hongyan Wang[1], Yunfeng Pan[1], Matthew D. Warren[1], Claire W. Hardy[1], Dong Liang[1], Elaine A. Liu[1], M. Hope Robinson[1], Soumon Rudra[1], Jie Wang[1], Shahrzad Ehdaivand[2], Mylin A. Torres[1], Ya Wang[1], and David S. Yu[1,*]

[1] Department of Radiation Oncology, Emory University School of Medicine, Atlanta, GA 30322 USA
[2] Department of Pathology, Emory University School of Medicine, Atlanta, GA 30322 USA

* Corresponding Author:
David S. Yu, M.D., Ph.D.
Department of Radiation Oncology
Emory University School of Medicine
1365 Clifton Rd NE, C3008
Phone: 404-778-1758, Fax: 404-778-5520
e-mail: dsyu@emory.edu

Inventory of Supplementary Information

Figure S1

Figure S2

Figure S3

Figure S4

Table S1

Table S2

Table S3

Supplementary Figure S1. Synthetic lethal screen controls. Western blot analysis demonstrating efficiency of ATR and CHK1 knockdown with indicated siRNAs in MDA-MB-231 cells.

Supplementary Figure S2. NEK9 depletion causes replication stress hypersensitivity. **(A)** MIA PaCa-2 cells were transfected with NT, ATR, or NEK9 siRNA, split 1:4 24 hours later, and treated 24 hours later with or without 5 *u*M gemcitabine for 72 hours prior to assaying for cell viability. Mean and standard deviation of gemcitabine treated to untreated cell viability relative to NT siRNA from three replicas is shown. * $p<0.05$, ** $p<0.01$, *** $p<0.001$. **(B)** Western blot analysis demonstrating efficiency of ATR and NEK9 knockdown with indicated siRNAs in MIA PaCa-2 cells. **(C)** HeLa cells were transfected with NT, ATR, or NEK9 siRNA, split 1:4 24 hours later, and treated 24 hours later with or without 5 *u*M gemcitabine for 72 hours prior to assaying for cell viability. Mean and standard deviation of gemcitabine treated to untreated cell viability relative to NT siRNA from three replicas is shown. * $p<0.05$, ** $p<0.01$.

Supplementary Figure S3. (A) NEK9 protein levels do not increase in S-phase. **(A)** MDA-MB-231 cells were treated with 500 μM mimosine for 24 hours, arresting them in G1 (t= 0 h). They were then washed and released into fresh media for the indicated times. DNA content was analyzed by flow cytometry, indicating cell populations mostly in S-phase (6 h), late S and G2/M (10 h), and G2/M and G1 (24 h). **(B)** Western blot analysis of lysate from MDA-MB-231 cells indicating expression level of NEK9 corresponding to the cell cycle profiles in **(A)**. **(C)** HEK 293T cells were treated with 500 μM mimosine for 24 hours, arresting them in G1 (t= 0 h). They were then washed and released into fresh media for the indicated times. DNA content was analyzed by flow cytometry, indicating cell populations mostly in S-phase (6 h), late S and G2/M (10 h), and G2/M and G1 (24 h). **(D)** Western blot analysis of lysate from HEK 293T cells indicating expression level of NEK9 corresponding to the cell cycle profiles in **(C)**.

Supplementary Figure S4. **(A)** NEK9 depletion does not impair ATR dependent phosphorylation of CHK1. HeLa cells were transfected with NT, CHK1, or NEK9 siRNA, and treated with 3 mM HU for 6 hours. Cell lysates were separated by SDS-PAGE, and immunoblotted with antibodies against NEK9, P-CHK1 Ser317, CHK1, and GAPDH. **(B)** NEK9 depletion impairs CHK1 kinase activity. CHK1 was purified from HeLa cells transfected with NT or NEK9 siRNA, treated with or without 3 mM HU for 6 hours, incubated in an *in vitro* kinase reaction with ^{32}P and CDC25C purified from bacterial cells as substrate, and processed by autoradiography. The reaction mixtures were separated by SDS-PAGE and immunoblotted with antibodies against NEK9 and GAPDH.

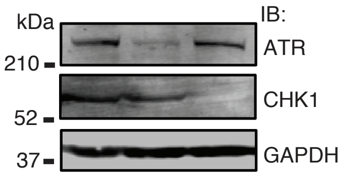

Supplemental Figure S1

A

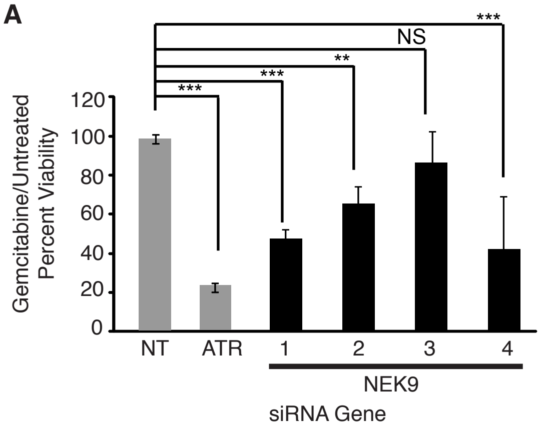

B

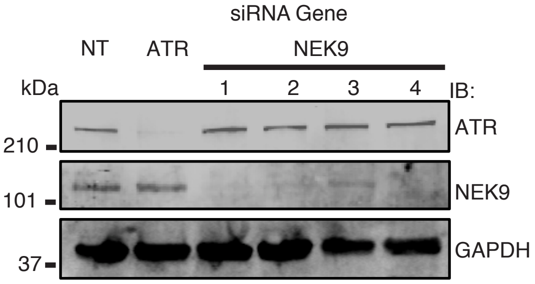

C

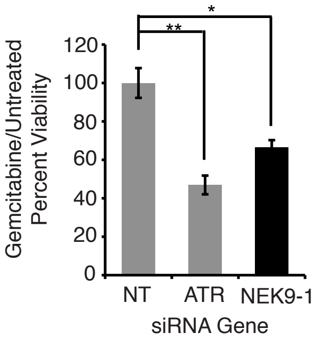

Supplemental Figure S2

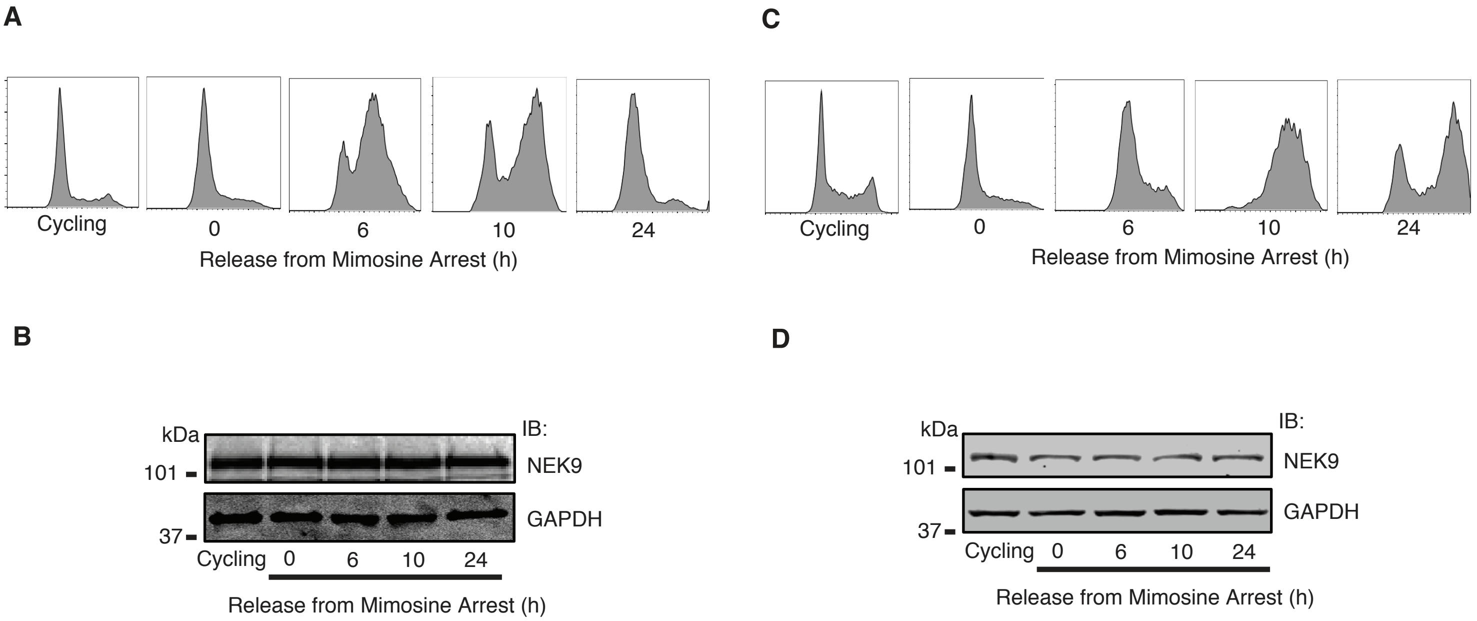

Supplemental Figure S3

A

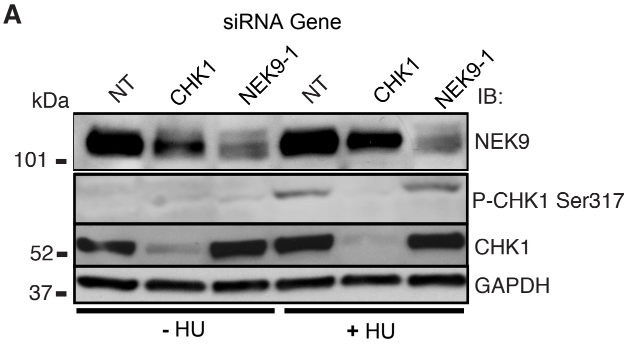

B

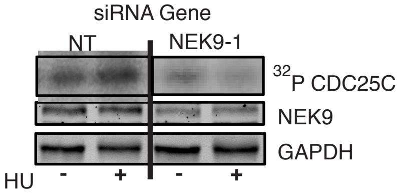

Supplemental Figure S4
